# Chemical Composition and Synergistic Potential of *Mentha pulegium* L. and *Artemisia herba alba* Asso. Essential Oils and Antibiotic against Multi-Drug Resistant Bacteria

**DOI:** 10.3390/molecules27031095

**Published:** 2022-02-07

**Authors:** Fahima Bekka-Hadji, Isabelle Bombarda, Ferhat Djoudi, Sofiane Bakour, Abdelaziz Touati

**Affiliations:** 1 Département de Microbiologie Appliquée et Sciences Alimentaires, Faculté des Sciences de la Nature et de la Vie, Université de Jijel, Jijel 18000, Algeria; fahima.bekka@hotmail.com; 2 Laboratoire d’Ecologie Microbienne, Faculté des Sciences de la Nature et de la Vie, Université de Bejaia, Bejaia 06000, Algeria; djoudi.ferhat@gmail.com (F.D.); ziz1999@yahoo.fr (A.T.); 3 Aix Marseille Univ, Université Avignon, CNRS, IRD, IMBE, 13013 Marseille, France; 4 Aix Marseille Univ, IRD, APHM, MEPHI, IHU-Méditerranée Infection, 13013 Marseille, France; b.sofiane-microbio@hotmail.fr

**Keywords:** essential oil, antibacterial activity, multi-drug-resistant bacteria, antibiotic, synergism

## Abstract

The essential oils were obtained by hydrodistillation from aerial parts of *Mentha pulegium* L. (*M*. *pulegium* L.) and *Artemisia herba alba* (*A. herba alba*) Asso. and analyzed by gas chromatography–flame ionization detector chromatograpy (GC–FID) and gaz chromatography–mass spectrometry (GC–MS). The antibacterial activities of the oils were determined by the disk diffusion method and a microdilution broth assay against six bacteria stains. The combinations of these essential oils with antibiotics were evaluated against two multi-drug-resistant bacteria strains: imipenem-resistant *Acinetobacter baumannii* (IRAB S3310) and methicillin-resistant *Staphylococcus aureus* (MRSA S19). The chemical analysis of *M*. *pulegium* essential oil revealed the presence of pulegone (74.8%) and neoisomenthol (10.0%). *A. herba alba* essential oil was characterized by camphor (32.0%), α-thujone (13.7%), 1,8-cineole (9.8%), β-thujone (5.0%), bornéol (3.8%), camphene (3.6%), and *p*-cymene (2.1%). All strains tested except *Pseudomonas aeruginosa* were susceptible to these oils. The combinations of essential oils with antibiotics exerted synergism, antagonism, or indifferent effects. The best effect was observed with *A. herba alba* essential oil in association with cefoxitin (CX) against MRSA S19. However, for IRAB S3310, the strongest synergistic effect was observed with *M. pulegium* in association with amikacin (AK). This study demonstrated that *M. pulegium* and *A. herba alba* essential oils have antibacterial activities which could be potentiated by antibiotics especially in the case of IRAB S3310.

## 1. Introduction

Antibiotics are one of the most common drug groups used in human and veterinary medicine. However, the massive and sometimes inappropriate use of antibiotics contributes to the emergence of multi-resistant bacteria including methicillin-resistant *Staphylococcus aureus* (*S. aureus*; MRSA) and carbapenems-resistant *Acinetobacter baumannii* (CRAB), which are considered to be the most multi-resistant pathogens feared in nosocomial infections [1,2]. The resistance of *S. aureus* to antibiotics is common. The capacity to cause various infections and its adaptation ability are the main concerns about this highly virulent pathogen. In addition, infections due to the antibiotics-resistant acinetobacter, including imipenem (IMP), have emerged over the years, leading up to therapeutic impasses. The growing concern about drug resistance has led researchers to focus more attention on natural products, including plants, with antimicrobial properties as a promising source of antimicrobial agents [3]. The antimicrobial properties of essential oils (EOs) from a wide variety of aromatic plants have been assessed and reviewed and confirm their use in traditional medicine as well as to extend the shelf life of foods [4,5,6,7,8].

Studies have shown the antibacterial activity of EOs against multi-resistant Gram-negative bacteria including *Escherichia coli* (*E. coli*) resistant to nalixidic acid or *Klebsielle pneumoniae*, a β-lactamase-producing strain with a widened CTXM spectrum [9,10] and Gram-positive bacteria, particularly against methicillin-resistant *S. aureus* [11,12,13,14]. The metabolite diversity of the oils confers a wide variety of biological properties, some of which constitute alternatives or supplements to synthetic compounds [15]. The synergistic effect obtained from a combination of antibiotics with plant extracts against resistant bacteria leads to new ways for the treatment of infectious diseases [16]. At the same time, most in vitro studies have focused on the combination of antibiotics and EOs to overcome the resistance problem [15,17].

The *Mentha* genus is a member of the *Labiatae* family. Leaves, flowers and stems of *Mentha* sp. are frequently used in herbal teas or as additives in commercial spice mixtures for many foods to offer flavor. In addition, *Mentha* sp. is often used in folk medicine to treat various diseases such as nausea, bronchitis, and ulcerative colitis.

*Mentha pulegium* L. (*M. pulegium* L.) is one of the *Mentha* species commonly known as pennyroyal. This species is generally known under the name ‘‘Feliou”. The chemical composition of *M. pulegium* EO has been subjected to several studies, which have shown differences in their constituents depending on the country and region of cultivation [18,19,20].

The genus *Artemisia* (more than 400 species) belongs to the *Asteraceae* family. It is common in the North and East Africa, but it also described in the Southern Hemisphere [21,22]. In Algeria, approximately 12 species are listed including *Artemisia herba alba* (*A. herba alba*) called Echih in Arabic [18]. This species is used to treat ulcers, dyspepsia, liver disorders, mouth ulcers, fungal infections, insect bites and scorpion, and all forms of poisoning [22,23]. It enjoys a great popularity, probably based on its antiseptic, antispasmodic, and antitumor properties, and the EO of this species is used primarily in perfumery and cosmetics [22,23].

This study aimed to evaluate the chemical compositions and the antibacterial activities of these two EOs, *M. pulegium* L. and *A. herba alba* Asso., growing in Bejaia (Algeria) and largely used in Algerian folk medicine, against a large panel of Gram-positive and Gram-negative bacterial strains including two multi-drug bacteria (methicillin-resistant *S. aureus* and imipenem-resistant *Acinetobacter baumannii*). In a second step, the associative effects of these two EOs with different groups of antibiotics against MRSA and IRAB isolates were studied for the first time.

## 2. Results and Discussion

### 2.1. EOs Chemical Compositions

The EO of *M. pulegium* studied was characterized by high contents of pulegone (74.8%) and menthol (10.0%). The complete relative composition was published in our previous work [13]. The high level of pulegone was never put in evidence and was characteristic of this algerian sample from Bejaia.

The EO of *A. herba alba* studied in this work contained camphor as a main product (32.0%), followed by α-thujone (13.7%), 1,8-cineole (9.8%), β-thujone (5.0%), borneol (3.8%), camphene (3.6%), and *p*-cymene (2.1%) (Table 1).

The study of a large number of samples from Maroc allowed [24] describing four chemotypes of which the differences mainly concern three ketones, α- and β-thuyone and camphor, representing between 60% and 80% of the total EO. The four chemotypes reported were α-thuyone/camphor, camphor, α-thuyone, and β-thuyone. Two other chemotypes including davanone and *cis*-chrysanthenyl acetate have also been identified [25]. Other chemotypes were reported from Algerian *A. herba alba* EOs including camphor, α-thuyone/β-thuyone, 1,8-cineole, and chrysanthenyl derivatives [26,27,28]. From all these works, our sample of *A. herba alba* can be classified in the camphor chemotype.

### 2.2. Antibacterial Activity

The antibacterial activities of *M. pulegium* and *A. herba alba* EOs against several bacteria were examined in the present study. The data obtained were expressed by the diameters of the inhibition zones and the minimal inhibitory concentrations (MICs). The results are given in Table 2 (Figures S1 and S2).

The results showed that the EOs of the two species had great potential for antibacterial activities against all bacterial strains tested, except *Pseudomonas aeruginosa* (*P. aeruginosa*). The diameters of inhibition zones and MIC values for bacterial strains, which were sensitive to the EO of *M. pulegium*, varied from 17.8 to 25.3 mm and from 1.2 to 9.4 µL/mL, respectively. For the EO of *A. herba alba,* the diameters of inhibition zones and MIC values varied from 13.2 to 19.7 mm and from 1.2 to 4.7 µL/mL, respectively.

Several authors have reported activity for the EO of *M. pulegium* against *S. aureus*, *Listeria monocytogenes* strains, and other Gram-negatives bacteria, except *P. aeruginisa* [20,29]. The antibacterial activity of this EO might be due to the presence of pulegone, menthone, and neo-menthol [30]. Duru et al. [31] demonstrated that pulegone exhibits a high antimicrobial activity on the all-test bacteria, especially *Salmonella typhimurium* and *S. aureus*.

The EO of *A. herba alba* tested in this study exhibited an interesting antibacterial activity against all strains except *P. aeruginosa*. The less sensitivity to *P. aeruginosa* strain was already observed for various chemotypes of *A. herba alba* [32].

Several authors report the low or even absence of the activity of EOs against *P. aeruginosa* [33,34]. The Iranian fresh oils of *Thymus persicus* L. and *Thymus kotschyanus* Boiss. were tested for their bacteriostatic and bactericidal effects at a dilution of 1/16 against five bacterial stains. *P. aeroginosa* is not affected with inhibition zones ranging from 6 to 12 mm (disc diameter included). However, for the *S. aureus* strain, the inhibition zone obtained is 75 mm [35].

### 2.3. Screening the Synergistic Effect of the EO with Antibiotic Discs

In this work, the synergy was studied between EOs (*M. pulegium* L. and *A. herba alba* Asso.) and a set of antibiotics belonging to different groups. The associations were studied on two multiresistant strains: imipenem-resistant *Acinetobacter baumannii* (IRAB S3310), which is resistant to imipenem (IMP), cefotaxime (CTX), cefipime (FEP), and other antibiotics, as well as methicillin-resistant *S. aureus* (MRSA S19), which showed a resistance towards oxacillin (OX) and cefoxitin (CX). 

The inhibitory effects of three antibiotics including OX, CX, and vancomycin (VAN) and the EOs of *M. pulegium* and *A. herba alba* against MRSA S19, alone and in combination, are presented in Table 3 and Table 4.

The values of the diameters of inhibition zones did not include the disc diameter (6 mm) in this part. A synergetic effect was observed for the two EOs and particularly in association with CX. The best effect was observed for the association of CX with *A. herba alba* EO against MRSA S19 (the inhibition zone diameter of the EO alone was 7.5 mm, and that was 29.0 mm in association). According to Uzair et al. [36], the amoxicillin antibacterial activity against MRSA can be enhanced using active constituents present in the EOs of *M. pulegium* and *A. herba alba* used in traditional medicine has been reported to have a significant effect against the resistance of *S. aureus* when combined with chloramphenicol, gentamicin, and cephalexin [37]. D’Arrigo et al. [38] showed that the EO of *Melaleuca alternifolia* has a synergistic activity against *S. aureus* when combined with tobramycin (TOB). The combination of thymoquinone and thymohydroquinone with antibiotics (ampicillin, cephalexin, chloramphenicol, tetracycline, gentamicin, and ciprofloxacin (CIP)) exerts synergism in *S. aureus*. On the other hand, in Gram-negative bacteria, synergism, antagonism, and indifferent effects were detected [39].

The antibacterial activities against imipenem-resistant *Acinetobacter baumannii* (IRAB S3310) conducted on various groups of antibiotic including amikacin (AK), nalidixic acid (NA), aztreonam (AT), ceftazidime (CAZ), ciprofloxacin (CIP), cefotaxime (CTX), cefepime (FEP), imipenem (IMP), piperacillin (PIP), ticarcillin–clavulanic acid (TCC), ticarcillin (TIC), and tobramicin (TOB) and the EOs of *M.* *pulegium* and *A. herba alba*, alone and in combination, are presented on Table 3 and Table 4 (and Figures S3 and S4). The combination of *M. pulegium* EO and different antibiotics showed antagonistic interactions with NA, AT, CIP, CTX, PIP, and TIC compared to the EO alone, while a synergistic effect was obtained with AK, CAZ, FEP, IMP, TCC, and TOB. The greatest effect was obtained for AK. In the case of *A. herba alba*, antagonistic interactions were observed for AK, AT, CIP, FEP, PIP, and TOB, while an indifferent effect was obtained with CAZ and TIC. Unlike the EO alone, synergistic effects were obtained with NA, CTX, IMP, and TCC, and the greatest effect was obtained for CTX.

The EO of *M. pulegium* had a better effect than the EO of *A. herba alba*. Strong synergistic effects were observed for *M. pulegium* EO in association with antibiotics belonging to penicillin, cephalosporin, carbapenems and aminoglycoside groups. In fact, the inhibition zone diameter increased from 19.3 mm for the oil alone to 27.5 mm for the EO/TCC combination and to 34 mm for the EO/AK combination. According to the results cited above, we found that the sensitivity of IRAB S3310 to the EOs/antibiotics combinations tested differed according to the EO and the antibiotic used in combination. The EO of *M. pulegium* was the most active in combination with the different antibiotics; the combination with antibiotics often gave a synergistic effect. However, the activity was less important for the EO of *A. herba alba*.

Rosato et al. [15] have underlined the strong synergy observed between gentamicin and *Anibarosae* *odora* against the Gram-negative *A.* *baumannii.* The combinations of coriander oil with chloramphenicol against *A. baumannii* have shown a synergistic effect. A synergistic, but less pronounced, behavior can be seen when combining coriander oil with either ciprofloxacin CIP, gentamicin, or tetracycline [17]. The study conducted by Boonyanugomol et al. [40] has shown that the EO of *Zingiber cassumunar* has a synergistic effect when combined with aminoglycosides, fluoroquinolones, and tetracyclines against the extensively drug-resistant *A.* *baumannii* strains.

The different activities observed for the EOs of *M. pulegium* and *A. herba alba* (synergy, antagonism, and indifference) may be explained by the mode of action of the different compounds of the EOs in combination with antibiotics. According to Rosato et al. [15], the mechanisms of action based on the synergism of gentamicin/EOs and in particular gentamicin/*Pelargonium graveolens* and gentamicin/*Anibarosae odora* are very difficult to elucidate. For this purpose, different hypotheses should be considered. All interactions between antimicrobial compounds can alter the effectiveness, and synergistic or antagonistic relationships may result in competition for possible primary targets. On the other hand, a synergistic multi-target effect could occur by involving enzymes, substrates, metabolites and proteins, receptors, ion channels, transport protein, ribosomes, DNA/RNA, and physicochemical mechanisms.

It also found that some antibiotics that are ineffective against *A. baumannii* show very interesting synergistic activities, which can be explained by the fact that the molecule contained in this antibiotic helps to lead the EO to its target. The action of tea tree EO did not cause either the leakage of potassium ions in *P. aeruginosa* or the release of absorbent molecules at 280 nm.

For this, research focused on how the microorganism tolerates high concentrations of this oil and/or components. These studies showed that tolerance is associated with the external membrane. The treatment of *P. aeruginosa* with nonapeptide B polymixin or EDTA causes the permeabilization of the membrane. As a result, the cells become more susceptible to bactericidal effects of tea tree EO, terpinen-4-ol, and γ-terpinene [41,42].

## 3. Materials and Methods

### 3.1. Plant Material and Isolation of EOs

Samples were collected in the north-eastern part of Algeria, in the Bordj-Mira city (*M. pulegium* L.) (36°33′52.9″ N; 5°17′11.7″ E) and in the city of Semaoun (*Artemisia herba alba* Asso.) (36°35′54.82″ N; 4°50′01.48″ E) in the district of Bejaia. The taxonomic identity of the plants was performed by Professor BEKDOUCHE F. (Batna 2 University, Algeria) by comparing voucher specimens with those of known identity already deposited in the herbarium of the Department of Botany, National Institute of Agronomy, Algeria. Aerial parts were dried at room temperature for 15 days.

Dried aerial parts were subjected to hydrodistillation for 3 h using a Clevenger-type apparatus. The EOs were collected after decantation, dried over anhydrous sodium sulfate to remove residual water and stored in sealed dark glass vials at 4 °C until used. The oil yields were 1.9% and 0.7% (*w*/*w* from the dried material) for *M. pulegium* and *A. herba alba*, respectively.

### 3.2. Gas Chromatography–Flame Ionization Detector (GS–FID) and Gas Chromatography–Mass Spectrometry (GC–MS) Analysis

An Agilent technologies 7890A gas chromatograph equipped with an FID was used for compound separations with an HP5 capillary column (30 m × 0.32 mm i.d., 0.40 μm phase thickness). The oven temperature program was as follows: first 2 min at 80 °C, then from 80 to 200 °C with an increasing rate of 5 °C/min, then 5 min at 200 °C, then from 200 to 260 °C with an increasing rate of 20 °C/min, and finally held at the final temperature for 5 min. The detector and inlet temperatures were 280 °C. Hydrogen was used as a carrier gas at a constant flow rate of 1 mL/min with a split ratio of 70/1. The injections were 1 μL of EO in methylene chloride (50 mg of EO in 1 mL CH_2_Cl_2_).

The GC–MS analyses were performed using an Agilent technologies 7890A gas chromatograph equipped with an HP5MScapillary column (30 m, 0.25 mm, 0.25 µm), and a mass detector MS 5975C VL MSD was operated in the EI mode. Helium was used as a carrier gas at a flow rate of 1 mL/min with a split ratio of 50/1. The oven temperature program was as follows: first 2 min at 80 °C, then from 80 to 200 °C with an increasing rate of 5 °C/min, then 5 min at 200 °C, then from 200 to 260 °C with an increasing rate of 20 °C/min, and finally held at the final temperature for 5 min. The detector and inlet temperatures were 280 °C. The identification of components was based on the comparison of their mass spectra with those of WILEY and NIST Libraries as well as on the comparison of their retention indices of the authentic standard with the literature [43].

### 3.3. Tested Bacterial Isolates

In this study, six bacterial strains were used. The bacterial cells assayed included three Gram-positive bacteria, i.e., *Listeria innocua* CLIP 74915, *S. aureus* ATCC25922, and methicillin-resistant *S. aureus* strain S19 (MRSA S19) (ST80-MRSA-IVk, PVL+) recovered from pus), and three Gram-negative bacteria, i.e., *E. coli* ATCC25922, *Pseudomonas aeruginosa* ATCC27853, and Imipenem-resistant *Acinetobacter baumannii* strain 3310 (IRAB S3310) isolated from the catheter, producing OXA-23 enzyme and resistant to cefotaxime (CTX) and cefepime (FEP). The bacterial strains were stored at 4 °C in a nutrient broth for the Gram-negative bacteria and in a brain heart infusion broth for the Gram-positive bacteria. Fresh cultures were prepared on a Mueller–Hinton Agar at 37 °C for 24 h.

### 3.4. Study of the Antibacterial Activities of EOs in Solid Media

The antibacterial activities of the EOs were tested according to the aromatogram method.

After the inoculation of the bacterial strain (load of 10^8^ Colony forming Unit (CFU)/mL), discs of Whatman No. 1 paper (6 mm in diameter) were deposited on an Mueller–Hinton Agar. Subsequently, 10 μL of each EO were placed on each disc. After incubation at 37 °C for 24 h, the diameters of the inhibition zones around the discs were measured. The tests were performed in triplicate, and the result was expressed as the mean of the three tests results ± standard deviation [13].

### 3.5. Study of the Antibacterial Activities of EOs in a Liquid Medium

The MICs of the EOs were determined using the Mueller–Hinton broth micro-dilution method [44]. The ranges of the EOs between 0.3 and 300 were performed in a 96-well round-bottomed microplate. All the tests were performed with an Mueller–Hinton broth added by tween 80 (0.5%) at a rate of 100 µL per well. Finally, each well was inoculated with 100 μL of the bacterial suspension (final load in each well is 5 × 10^5^ CFU/mL). After 24 h incubation at 37 °C, the MIC corresponded to the EO concentration of the first well showing no bacterial growth.

### 3.6. Screening the Synergistic Effect of the EO with Antibiotic Discs

The standard antibiotics used in this study included different groups of antibiotics: AK, NA, AT, CAZ, CIP, CTX, FEP, IMP, piperacillin (PIP), ticarcillin–clavulanic acid (TCC), ticarcillin (TIC), and TOB for IRAB S3310 and CX and OX for MRSA S19. The synergistic effect was tested according to the modified procedure of Boonyanugomol et al. [40]. The standard antibiotics discs were individually impregnated with 10 µL of each EO and placed on agar plates containing Mueller–Hinton Agar inoculated with a bacterial suspension (10^8^ CFU/mL). After incubation overnight, the zones of inhibition produced by the EOs combined with standard antibiotics were evaluated.

The data were interpreted as indifference, antagonism, and synergy by comparing the diameter of the inhibition zone in combination (EO + standard antibiotic) with the sum of the diameters of the inhibition zones of the two agents tested separately (EO and antibiotic).

## 4. Conclusions

The results of the screening of the associative effect of EOs of *M. pulegium* and *A. herba alba* with antibiotics in a solid medium showed that SARM S19 exhibited synergistic effects in association with CX with a better synergy in the case of *M. pulegium* EO. The result observed for the EOs with the different groups of antibiotics against the IRAB S3310 strain differed, depending on the EO and the antibiotic used (10 synergistic combinations, 12 antagonistic combinations, and two indifferent combinations). However, the strongest activity was observed for *M. pulegium* EO in combination with AK.

These preliminary results are promising in expanding the therapeutic arsenal of plants with antibacterial properties. Their screenings allow the discovery of new antibacterials, which may constitute an alternative to the use of conventional antibiotics that have become ineffective.

The results showed that EOs combined with antibiotics can be very effective sources to help fight against multi-resistant bacteria. In addition, the antagonistic effects observed indicated that all combinations are not recommended.

## Figures and Tables

**Table 1:** Chemical composition of *Artemisia herba alba* (*A. herba alba*) essential oil growing wild in Bejaia (Algeria).

RI ^a^	RI ^b^	Compounds	Relative Composition ^c^
913	924	α-thujene	0.3
924	932	α-pinene	1.6
945	946	camphene	3.6
974	969	sabinene	0.3
981	974	β-pinene	0.2
990	988	β-myrcene	0.3
1009	1002	α-phellandrene	0.1
1015	1008	3-carene	0.2
1020	1014	α-terpinene	0.2
1027	1020	*p*-cymene	2.1
1030	1024	limonene	0.4
1035	1026	1,8-cineole	9.8
1058	1054	γ-terpinene	0.7
1068	1065	*cis*-sabinene hydrate	0.4
1091	1083	fenchone	0.6
1107	1101	α-thujone	13.7
1118	1112	β-thujone	5.0
1128	1124	chrysanthenone	1.7
1143	1139	pinocarvéol	1.2
1151	1141	camphor	32.0
1169	1167	menthol	1.0
1172	1165	borneol	3.8
1183	1174	terpinen-4-ol	0.9
1191	1179	*p*-cymen-8-ol	0.2
1205	1195	myrtenal	0.5
1252	1239	carvone	0.2
1296	1289	thymol	0.6
1306	1298	carvacrol	1.7
1331	1324	myrtenyl acetate	0.4
1507	1500	bicyclogermacrene	0.2
1532	1522	δ-cadinene	0.1
1564	-	eudesma-3,7(11)-diene	0.1
1595	1582	caryophyllene oxide	0.2
1615	1602	ledol	0.1

^a^ Retention indices relative to homologous *n*-alkanes C6–C26 obtained on an HP-5 capillary column. ^b^ Retention indices from the literature (Adam, 2007). ^c^ Experimental relative area percentage obtained by chromatography (GC) on an HP-5 capillary column.

**Table 2:** IZDs (mm) and MICs for *Mentha pulegium* L. (*M. pulegium* L.) and *A. herba alba* Asso. essential oils against bacteria strains.

Plants	Essential Oil	*Staphylococcus aureus* ATCC25923	MRSA S19	*Listeria innocua* CLIP 74915	*Escherichia coli* ATCC25922	*Pseudomonas aeruginosa* ATCC27853	IRABS3310
*M. pulegium* L.	IZD	18.2 ± 0.4	17.8 ± 2.9	20.7 ± 1.6	17.2 ± 0.7	06.6 ± 0.5	25.3 ± 3
	MIC	1.2	2.3	1.2	9.4	75	1.2
*A. herba alba* Asso.	IZD	19.7 ± 0.7	13.5 ± 0.0	13.2 ± 0.9	13.2 ± 0.1	NZ	15.3 ± 0.6
	MIC	1.2	1.2	1.2	4.7	18.8	1.2
ciprofloxacin (CIP)	IZD	-	-	-	34 ± 2.8	38.5 ± 0.0	14 ± 0.0
amikacin (AK)	IZD	-	-	-	20 ± 0.0	26 ± 0.0	12 ± 0.5
oxacillin (OX)	IZD	25.5 ± 0.7	19 ± 0.0	-	-	-	-
cefoxitin (CX)	IZD	25.5 ± 0.7	19.5 ± 0.0	-	-	-	-
vancomycin (VAN)	IZD	18.8 ± 2.5	17.5 ± 0.0	-	-	-	-

IZD, the inhibition zone diameter (mm) including the disc diameter of 6 mm. The values are given as the mean ± standard deviation; NZ, no inhibition zone; MIC, minimal inhibitory concentration in µL/mL.

**Table 3:** IZDs ^a^ (mm) of *M. pulegium* essential oil and antibiotics, alone and in combination, against MRSA S19 and IRABS3310 bacteria.

Groups		Antibiotics	Inhibition Zone ^a^ with the Antibiotics	Inhibition Zone ^a^ with the Essential Oil	Sum of the Inhibitions Zone ^a^ with the Antibiotics and Essential Oil	Inhibition Zone ^a^ with the Antibiotics and the Essential Oil in Association	Effects ^b^
MRSA S19	penicillin	OX	13.0 ± 0.0	11.8 ± 2.9	>24.8	19.4 ± 1.5	A
	cephalosporines	CX	13.5 ± 0.0	11.8 ± 2.9	>25.3	26.8 ± 0.6	S
	glycopeptids	VAN	11.5 ± 0.0	11.8 ± 2.9	>23.3	14.5 ± 0.0	A
IRAB S3310	penicillin	TIC	NZ	19.3 ± 3	>19.3	16.1 ± 1.0	A
		PIP	NZ	19.3 ± 3	>19.3	14.0 ± 2.0	A
		TCC	NZ	19.3 ± 3	>19.3	27.5 ± 1.3	S
	cephalosporines	CTX	NZ	19.3 ± 3	>19.3	17.0 ± 2.0	A
		CAZ	NZ	19.3 ± 3	>19.3	22.7 ± 1.5	S
		FEP	NZ	19.3 ± 3	>19.3	24.5 ± 0.9	S
	carbapenems	IMP	NZ	19.3 ± 3	>19.3	24.8 ± 0.3	S
	aminoglycosides	AK	6.0 ± 0.0	19.3 ± 3	>25.3	34.0 ± 1.0	S
		TOB	9.0 ± 0.0	19.3 ± 3	>28.3	29.0 ± 3.0	S
	fluoroquinolones	CIP	8.0 ± 0.0	19.3 ± 3	>27.3	24.7 ± 0.6	A
		NA	NZ	19.3 ± 3	>19.3	18.5 ± 1.3	A
	monobactames	AT	4.0 ± 0.0	19.3 ± 3	>23.3	16.8 ± 1.8	A

^a^ The inhibition zone non includes the diameter of the disc, and values are given as the mean ± standard deviation; ^b^ A, antagonism; S, synergism; NZ, no inhibition zone; OX, oxacillin; CX, cefoxitin; Van, vancomycin; TIC, ticarcillin; PIP, piperacillin; TCC, ticarcillin–clavulanic acid; CTX, cefotaxime; CAZ, ceftazidime; FEP, cefepime; IMP, imipenem; AK, amikacin; TOB, tobramicin; CIP, ciprofloxacin; NA, nalidixic acid; AT, aztreonam.

**Table 4:** IZDs ^a^ (mm) of *A. herba alba* essential oil and antibiotics, alone and in combination, against MRSA S19 and IRABS3310 bacteria.

Groups		Antibiotics	Inhibition Zone ^a^ with the Antibiotics	Inhibition Zone ^a^ with the Essential Oil	Sum of the Inhibitions Zone ^a^ with the Antibiotics and the Essential Oil	Inhibition Zone ^a^ with the Antibiotics and the Essential Oil in Association	Effects ^b^
**MRSA S19**	penicillin	OX	13.0 ± 0.0	07.5 ± 0.0	>20.5	20.8 ± 0.6	I
	cephalosporines	CX	13.5 ± 0.0	07.5 ± 0.0	>21.0	29.0 ± 0.0	S
	glycopeptids	VAN	11.5 ± 0.0	07.5 ± 0.0	>19.0	17.7 ± 0.3	A
**IRABS3310**	penicillin	TIC	**NZ**	9.3 ± 0.6	>09.3	09.6 ± 2.1	I
		PIP	**NZ**	9.3 ± 0.6	>09.3	06.6 ± 1.3	A
		TCC	**NZ**	9.3 ± 0.6	>09.3	10.5 ± 0.5	S
	cephalosporines	CTX	**NZ**	9.3 ± 0.6	>09.3	13.3 ± 2.6	S
		CAZ	**NZ**	9.3 ± 0.6	>09.3	09.6 ± 2.1	I
		FEP	**NZ**	9.3 ± 0.6	>09.3	08.8 ± 1.0	A
	carbapenems	IMP	**NZ**	9.3 ± 0.6	>09.3	10.8 ± 1.6	S
	aminoglycosides	AK	6.0 ± 0.0	9.3 ± 0.6	>15.3	13.0 ± 2.6	A
		TOB	9.0 ± 0.0	9.3 ± 0.6	>18.3	17.0 ± 2.0	A
	fluoroquinolones	CIP	8.0 ± 0.0	9.3 ± 0.6	>17.3	15.3 ± 1.0	A
		NA	NZ	9.3 ± 0.6	>09.3	10.3 ± 1.5	S
	monobactames	AT	4.0 ± 0.0	9.3 ± 0.6	>13.3	09.5 ± 3.3	A

^a^ The inhibition zone non includes the diameter of the disk, and values are given as the mean ± standard deviation; ^b^ A, antagonism; S, synergism; NZ, no inhibition zone; OX, oxacillin; CX, cefoxitin; VAN, vancomycin; TIC, ticarcillin; PIP, piperacillin; TCC, ticarcillin–clavulanic acid; CTX, cefotaxime; CAZ, ceftazidime; FEP, cefepime; IMP, imipenem; AK, amikacin; TOB, tobramicin; CIP, ciprofloxacin; NA, nalidixic acid; AT, aztreonam.

## Data Availability

The study did not report any data.

## Supplementary Materials

The following supporting information can be downloaded. Figure S1: Effect of the *Mentha pulegium* essential oil against bacterial strains; Figure S2: Effect of the *Artemisia herba alba* essential oil against bacterial strains; Figure S3: antibiogram showing the resistance of the strain IRAB S3310 against different antibiotics; Figure S4: Effect of the association of *Mentha pulegium* essential oil with amikacin (AK) and tobramicin (TOB) and *Artemisia herba alba* essential oil with cefotaxime (CTX) against IRAB S3310.

## Sample Availability

Samples of the compounds are not available from the authors.

## Abreviations

*A. baumannii*	*Acinetobacter baumannii*
*A. herba alba*	*Artemisia herba alba*
A	antagonism
AK	amikacin
AT	aztreonam
CAZ	ceftazidime
CIP	ciprofloxacin
CTX	cefotaxime
CX	cefoxitin
*E. coli*	*Escherichia coli*
EI	electronic impact
EO	essential oil
FEP	cefepime
GC–FID	gas chromatography–flame ionization detector
GC–MS	gas chromatography–mass spectrometry
i.d.	internal diameter
IMP	imipenem
IZD	inhibition zone diameter
*L. innocua*	*Listeria innocua*
MIC	minimal inhibitory concentration
*M. pulegium*	*Mentha pulegium*
NA	nalidixic acid
NZ	no inhibition Zone
OX	oxacillin
*P. aeruginosa*	*Pseudomonas aeruginosa*
PIP	piperacillin
*S. aureus*	*Staphylococcus aureus*
S	synergism
TCC	ticarcillin–clavulanic acid
TIC	ticarcillin
TOB	tobramicin
VAN	vancomycin
