# Supplementary material for: Chemical Composition and Synergistic Potential of Mentha pulegium L. and Artemisia herba alba Asso. Essential Oils and Antibiotic against Multi-Drug Resistant Bacteria
Source: Molecules. 2022 Feb 7;27(3):1095. doi: 10.3390/molecules27031095 (PMC8839733; doi:10.3390/molecules27031095)
Supplement: Supplementary file 1 [file molecules-27-01095-s001.zip › molecules-1555564-supplementary.pdf]

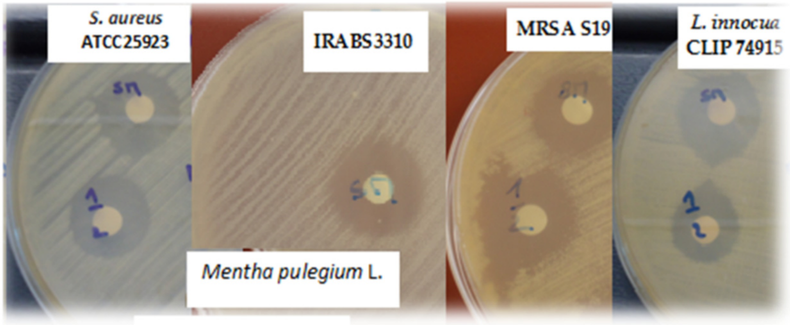

Figure S1 : effect of the *M. pulegium* essential oil against bacterial strains

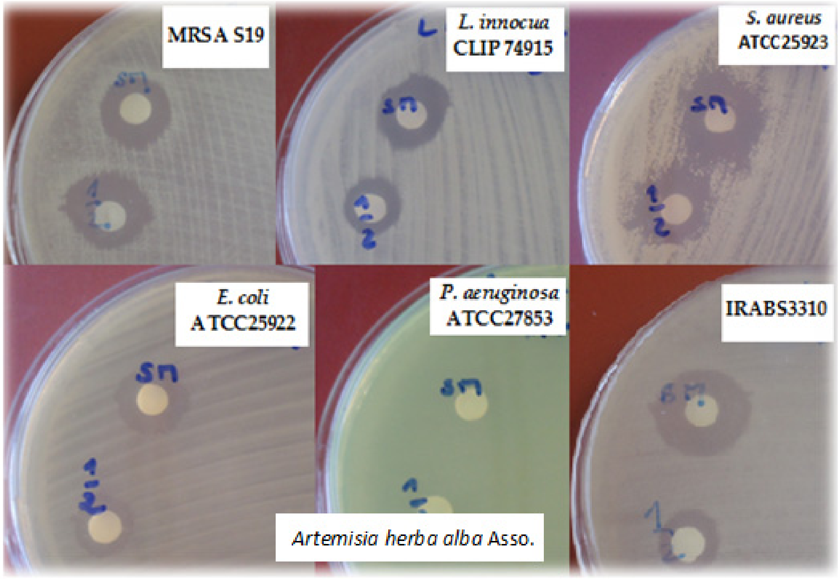

Figure S2 : effect of the *A. herba alba* essential oil against bacterial strains

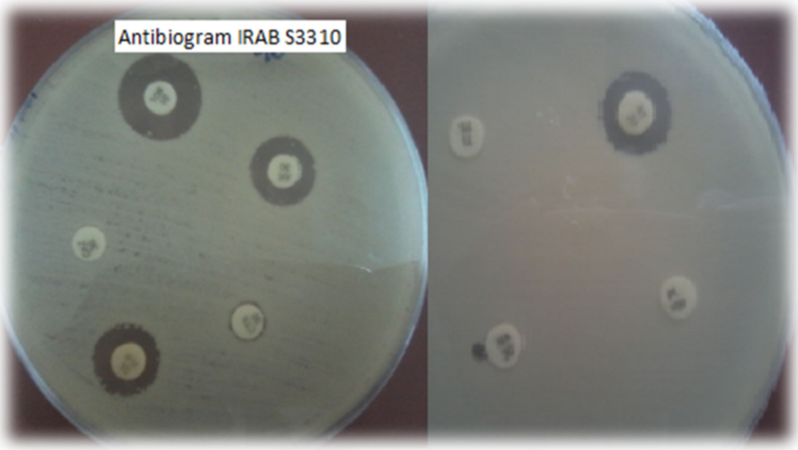

Figure S3 : antibiogram showing the resistance of the strain IRABS3310 against different antibiotics.

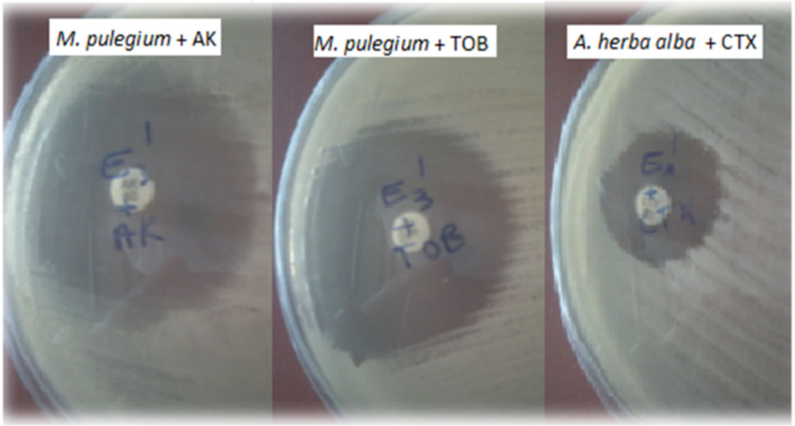

Figure S4 : Effect of the association of *M. pulegium* essential oil with amikacin (AK) and tobramicin (TOB) and *A. herba alba* essential oil with cefotaxime (CTX) against IRAB S3310
